# Makorin 1 is required for Drosophila oogenesis by regulating insulin/Tor signaling

**DOI:** 10.1371/journal.pone.0215688

**Published:** 2019-04-22

**Authors:** Eui Beom Jeong, Seong Su Jeong, Eunjoo Cho, Eun Young Kim

**Affiliations:** 1 Neuroscience Graduate Program, Department of Biomedical Sciences, Ajou University Graduate School of Medicine, Suwon, Kyunggi-do, Republic of Korea; 2 Department of Brain Science, Ajou University School of Medicine, Suwon, Kyunggi-do, Republic of Korea; Inha University, REPUBLIC OF KOREA

## Abstract

Reproduction is a process that is extremely sensitive to changes in nutritional status. The nutritional control of oogenesis via insulin signaling has been reported; however, the mechanism underlying its sensitivity and tissue specificity has not been elucidated. Here, we determined that *Drosophila* Makorin RING finger protein 1 gene (*Mkrn1*) functions in the metabolic regulation of oogenesis. *Mkrn1* was endogenously expressed at high levels in ovaries and *Mkrn1* knockout resulted in female sterility. *Mkrn1*-null egg chambers were previtellogenic without egg production. FLP-FRT mosaic analysis revealed that *Mkrn1* is essential in germline cells, but not follicle cells, for ovarian function. As well, AKT phosphorylation via insulin signaling was greatly reduced in the germline cells, but not the follicle cells, of the mutant clones in the ovaries. Furthermore, protein-rich diet elevated Mkrn1 protein levels, without increased mRNA levels. The p-AKT and p-S6K levels, downstream targets of insulin/Tor signaling, were significantly increased by a nutrient-rich diet in wild-type ovaries whereas those were low in *Mkrn1*^*exS*^ compared to wild-type ovaries. Taken together, our results suggest that nutrient availability upregulates the Mkrn1 protein, which acts as a positive regulator of insulin signaling to confer sensitivity and tissue specificity in the ovaries for proper oogenesis based on nutritional status.

## Introduction

Reproduction is an energetically expensive process because it involves massive cell proliferation and growth that requires synthesis of various proteins. Because of this high-energy demand, reproduction is tightly regulated by nutrient availability to ensure that reproduction only happens when there is a surplus of nutrients. When nutrients are scarce, reproduction is compromised to preserve energy for survival. Reproductive pauses in response to starvation are common among species of the animal kingdom; this reproductive dormancy can be quickly reversed by nutritional status [1–3].

*Drosophila* ovaries are composed of ovarioles that consist of sequentially developing egg chambers [4]. At the anterior end of an ovariole, germline stem cells reside in a structure called the germarium and proliferate to produce 16 interconnected germline cells, one of which becomes an oocyte while the remaining 15 cells become nurse cells. Groups of interconnected germline cells are called as germline cysts and newly produced germline cysts are enclosed by follicle cells and develop separately. Oogenesis can be roughly divided into 14 stages that can each be characterized by size and morphology. While germline cells do not divide after 16 cells, follicle cells go through mitosis until stage 6 and switch to endocycle stage (ME switch), where only genome replications occur. Notch signaling controls this ME switch [5, 6]. The ME switch is nutrient dependent and requires the *Foxo* gene [7]. After the ME switch, vitellogenesis, the yolk formation process by oocyte uptake of precursor proteins, and oocyte maturation occur. Whether oogenesis should continue or not is determined at a mid-oogenesis check point, which occurs right after the ME switch and before the vitellogenesis. The chief determining factor for oogenesis progression is if there are enough nutrients for reproduction. Under starvation conditions, programmed cell death occurs at stage 8 egg chambers [8].

The hormonal system of organisms relays nutritional information to organs. Insulin and insulin-like growth factor signaling is one of the most important hormonal systems for nutritional regulation. Insulin is released upon nutrient uptake and subsequently regulates processes that require energy, such as cellular growth, metabolism, and reproduction [9, 10]. Female reproductive organs rely on the insulin signaling system for proper function and to ensure that oogenesis occurs only when there are enough nutrients available [10]. The insulin signaling pathway and its role in ovarian development is conserved among species [11]. In flies, insulin receptor (*InR*) and insulin substrate protein 1 (*chico*) genetic mutation cause female sterility [12, 13]. Also, ovarian cells require the insulin signaling pathway for nutrient-dependent growth and vitellogenesis [8]. Insulin signaling also affects mammalian oocyte growth and development. In humans, ovarian malfunctions have been associated with defects in insulin signaling, such as insulin resistance [14, 15]. However, the mechanisms by which insulin regulates oocyte development have not been well established *in vivo*, despite evidence that insulin is involved in androgen production, gonadotropin signaling, PCOS (Polycystic Ovary Syndrome), and obesity-induced infertility [16].

Nutrient availability has been shown to delay or accelerate reproductive capabilities [17, 18]. Therefore, it can be inferred that ovaries possess a mechanism to distinguish quantitative differences in nutrient levels. We wanted to know how this quantitative nutritional information is interpreted at the molecular level and postulated that the strength of the nutrition-dependent regulatory signaling, such as insulin signaling, is directly influenced by the amount of nutrients available. Moreover, systemic insulin signaling is variable and tissue-specific because vital organs are preferentially spared from starvation. Indeed, ovaries are the most sensitive organ to reduced insulin signaling. In flies, mutation of *InR*^*E19*^, *lnk*^*4Q3/6S2*^, and *chico*^*1*^ cause reduction, but not complete abolition, of insulin signaling without exhibiting defects in vital organs; however, oogenesis is greatly compromised [12, 13, 19]. Mutation of *Irs-2* in mice also causes female sterility with other major defects [20]. Furthermore, it has been shown that mouse ovaries remain sensitive to insulin signaling while the pituitary gland becomes insulin resistant in a diet-induced obesity model [21]. However, the mechanism of tissue specificity and sensitivity of insulin signaling remains poorly understood.

The target of rapamycin (TOR) signaling pathway is another highly conserved nutrient sensing signal that is required for cell growth and proliferation [22, 23]. While insulin signaling senses and delivers systematic nutrient information, TOR signaling senses cellular amino acid levels and regulates anabolic processes. Insulin and TOR signaling interact to ensure cells integrate nutrient information from difference sources and perform appropriate cellular processes. In *Drosophila*, the ovaries require TOR activity for the proliferation and maintenance of germline cells [22]. Hence, interactions between the insulin and TOR signaling pathways are the most evident in ovaries. In *Drosophila*, PRAS40, the inhibitor of TOR signaling pathway, affects the interaction of the insulin and TOR signaling only in ovaries [23]

Steroid hormone signaling is also important for reproduction. In *Drosophila*, ecdysone, a steroid hormone that controls molting in insects, is required for reproductive processes. Developing egg chambers degenerate without ecdysone signaling [24, 25]. In general, steroid signaling is influenced by the nutritional and metabolic status of an organism. For example, nutrition affects ecdysone concentrations and InR mutation impairs ecdysone synthesis in *Drosophila* [26, 27]. In mammals, ovarian folliculogenesis is regulated by the interplay of hormones such as gonadotropins, steroids and insulin-like growth factors [28–30].

*Mkrn1* is a *Drosophila* homologue of mammalian makorin family and encodes RING zinc finger proteins with ubiquitin ligase activity. In mammals, Mkrn1 reportedly destabilizes many substrates including p53, p21, PPARγ, hTERT, PTEN, APC, and AMPK [31–36], and is therefore implicated in various cellular events. In our previous study, we showed that *Drosophila Mkrn1* is involved in the proper timing of the larval to pupal transition and affects final body size by regulating ecdysone synthesis in the prothoracic gland suggesting *Mkrn1* plays a role in the transition from growth to maturation [37]. Here, we found that female *Mkrn1* null mutant (*Mkrn1*^exS^) flies are sterile, and further demonstrated that *Mkrn1* is required for *Drosophila* oogenesis. Furthermore, *Mkrn1* was strongly expressed in ovaries and was upregulated by a protein-rich diet. *Mkrn1* null female flies exhibited vitellogenesis failure, which also occurs when flies were starved or nutrient signaling was perturbed. Insulin/Tor signaling was greatly reduced in *Mkrn1*^exS^ ovaries, implying a role as a positive regulator in the insulin/Tor signaling pathway. Collectively, our data support the notion that *Mkrn1* functions as a tissue-specific regulator of the insulin/Tor signaling pathway to activate oogenesis in ovaries in a nutrient-sensitive manner.

## Materials and methods

### Drosophila strains and culture conditions

The *Mkrn1* and *Mkrn1*^*exS*^ mutant *Drosophila* strains were generated as previously described [37]. Two lines with *Mkrn1* gene deletions—*Df(3L)BSC418/TM6C* (BDSC 24922) and *Df(3L)BSC419/TM6C* (BDSC 24923)—were used in this study. *NRE-EGFP* (BDSC 30727) was used to examine Notch signaling and *PRAS40*^*KO*^ (BDSC 76339) was used to examine epistatic interactions with *Mkrn1*^*exS*^.

All *Drosophila* stocks were maintained on a standard cornmeal food at room temperature except for the experimental groups that required nutrient-poor and -rich diet conditions. The nutrient-poor diet contained 10% sucrose and 2% agar, whereas the nutrient-rich diet contained 10% sucrose, 2% agar, and yeast paste. Females were cultured for 1 day under different nutritional conditions less than 8 hours after eclosion.

### Generation and analysis of mosaic clones

For FLP-FRT mosaic analysis, the *FRT80B Mkrn1*^*exS*^ strain was generated by *FRT80B* and *Mkrn1*^*exS*^ recombination followed by crossing with *hsflp; FRT80B*, *ubi-GFP* females. F1 generation, one-day-old, female flies were heat-shocked for 1 hr at 37°C twice a day for 3 consecutive days and kept in a 25°C incubator. Ovaries were dissected and subjected to immunostaining 10 days after the first shock.

### Quantitative real-time PCR

Adult female heads, thoraxes, abdomens, and ovaries were dissected. From these tissues, total RNA was isolated using QIAzol lysis reagent (QIAGEN) and reverse-transcribed with oligo-dT primers using Prime Script reverse transcriptase (TAKARA). Quantitative real-time PCR was performed using a Rotor Gene 6000 (QIAGEN) with SYBR Premix Ex Taq (Tli RNase H Plus; TAKARA). The following primers were used: *Mkrn1*-forward, 5’-GACGTGCGGCATCTGCTTTG-3’; *Mkrn1*-reverse, 5’-TGTTTGGCCTGACGCCATGT-3’; *Phm*-forward, 5’-GCTTGCATTTCCGAGACGAT-3’; *Phm*-reverse, 5’-ACGATCATCGAACCACCCTT-3’; *E74*-forward, 5’-CAAACCGAAGCTGGAGATGG-3’; and *E74*-reverse, 5’-TCGTCCACTTGATGAAACGC-3’. *Cbp20* or *actin* mRNA levels were used to normalize gene expression levels using the following primer sequence: *cbp20*-forward, 5’-GTATAAGAAGACGCCCTGC-3’; *cbp20*-reverse, 5’-TTCACAAATCTCATGGCCG-3’; *actin*-forward, 5’-CATGTTTGAGACCTTCAACACCCC-3’; *actin*-reverse, 5’-GCCATCTCCTGCTCGAAGTCTAG-3’. Data were analyzed and quantified using Rotor Gene 6000 software.

### Immunoblot analysis

We used our previously generated antibody against *Drosophila Mkrn1* [37]. Protein extracts from the head, thorax, abdomen, and ovaries of adult female *Drosophila* were prepared using lysis buffer (10 mM HEPES [pH 7.5]; 50 mM KCl; 10% glycerol; 5 mM Tris-HCl [pH 7.5]) with freshly added 5 mM EDTA, 1 mM DTT, 0.1% Triton X-100, protease inhibitor (Sigma), 1 mM Na_3_VO_4_, and 0.25 mM NaF (final concentration). For p-AKT and p-S6K analysis, phosphatase inhibitor cocktail 2 and 3 (Sigma) were used instead of Na_3_VO_4_ and NaF. The protein extracts were resolved by SDS-PAGE and the resulting blots were probed using the following primary antibodies: rabbit anti-Mkrn1, 1:3000 [37]; rabbit anti-phospho-AKT (Ser505), 1:1000 (Cell Signaling Technology, 4054); rabbit anti-AKT, 1:1000 (Cell signaling Technology, 9272); rabbit anti-phospho-S6K (Thr398), 1:1000 (Cell Signaling Technology, 9209); guinea pig anti-dS6K, 1:3000 [38]; mouse anti-Armadillo, 1:1000 (DSHB, N2 7A1); rabbit anti-p44/42 MAPK (Erk1/2), 1:2000 (Cell Signaling Technology, 9102). Band intensities were quantified using ImageJ software.

### Immunostaining of ovaries and image analysis

The ovaries of female adult flies were dissected in PBS; and approximately twenty ovaries for each sample were analyzed by immunostaining. The ovaries were fixed in 4% paraformaldehyde and rinsed with 0.5% PBST solution (1× PBS containing 0.5% Triton X-100). The fixed ovaries were incubated for 1 hr in blocking solution comprised of 0.5% PBST and 10% horse serum. Primary antibodies were diluted in blocking solution and incubated with samples overnight at 4°C followed by washing with 0.5% PBST. The samples were subsequently incubated with secondary antibodies diluted in blocking solution overnight at 4°C. The ovaries were washed and stained with hoechst33342 (Sigma, 1:1000), mounted on slides, and incubated with Phalloidin-TRITC (Sigma, P1951) in PBS (2.5 ug/ml) for 30 min to stain the actin filaments. Confocal images were obtained with a LSM710 or LSM800 Microscope (Zeiss) and processed with Zen software (Zeiss). The following antibodies and final dilutions were used: rabbit anti-Mkrn1, 1:3000 [37]; goat anti-VASA, 1:500 (Santa Cruz, sc-26877); mouse anti-Cut, 1:100 (DSHB, 2B10); mouse anti-Hindsight, 1:100 (DSHB, 1G9); mouse anti-Broad Core, 1:100 (DSHB, 25E9.D7); rabbit anti-phospho-AKT (Ser473), 1:100 (Cell Signaling Technology, 4060), Alexa 555-conjugated goat, anti-mouse and goat, anti-rabbit IgG, 1:100 and 1:200, respectively (Sigma); Alexa 488-conjugated donkey, anti-goat IgG, 1:200 (Sigma).

### Rapamycin treatment

Rapamycin (Sigma) was dissolved in ethanol and diluted to a final concentration of 1 μM in PBS. Ovaries were dissected from female files 6 to 8 hours after eclosion and incubated in PBS with or without rapamycin for 1 hour, or 2 hours. After incubation, the ovaries were immediately processed for western blot analysis.

## Results

### *Mkrn1* expression was enriched in Drosophila ovaries and *Mkrn1*-null females were sterile

**Fig 1:**
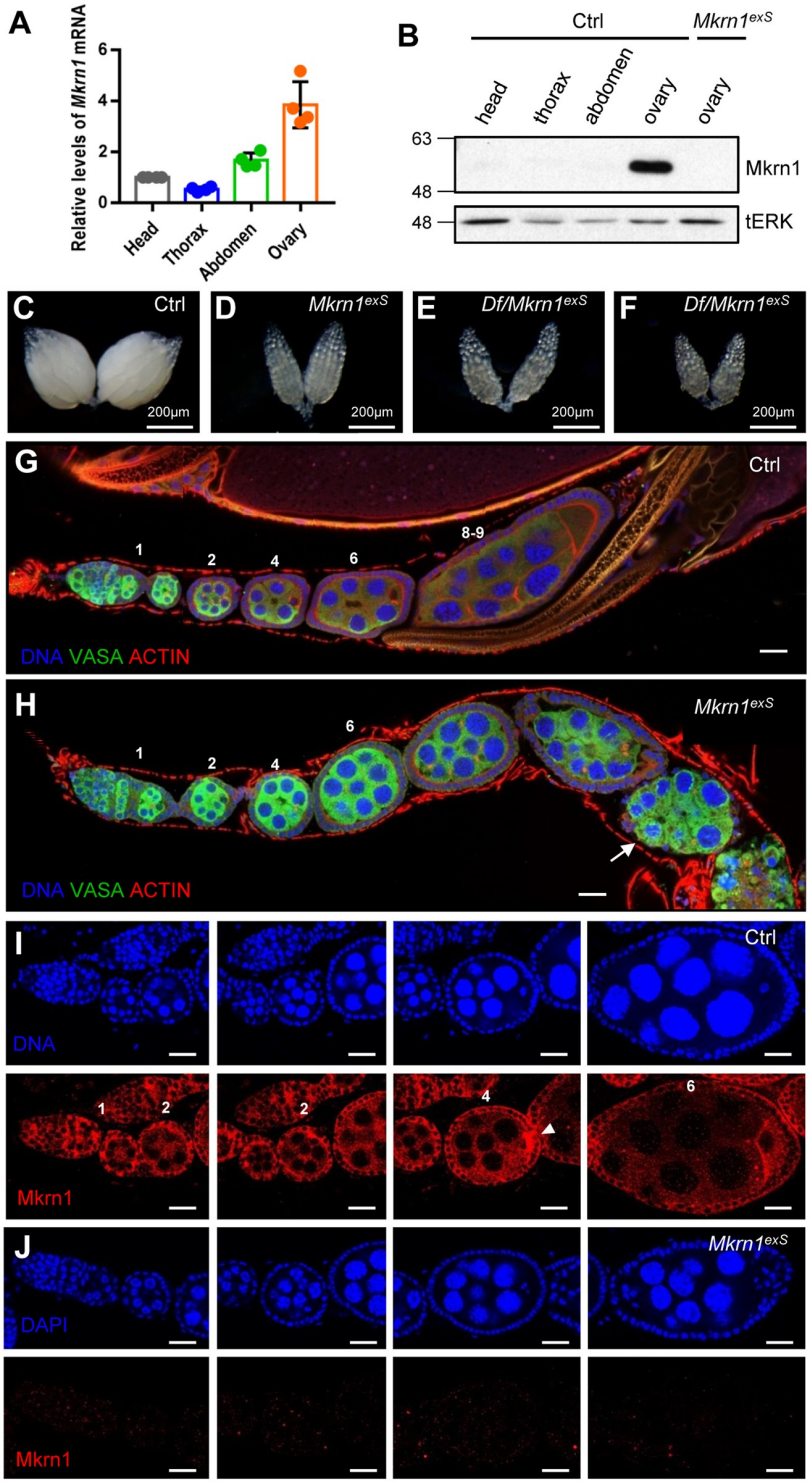
*Mkrn1* expression was highly enriched in control ovaries and *Mkrn1*-null ovaries exhibited a phenotype. (A) One-day-old, control female flies from standard nutrient conditions were dissected for baseline analyses. Total mRNA was extracted from the heads, thoraxes, abdomens, and ovaries and analyzed by quantitative real-time PCR. Relative mRNA levels for *Mkrn1* and reference gene *Actin* are shown. Error bars represent the SEM from four independent experiments. (B) Protein extracts from indicated tissues were analyzed by immunoblot using the anti-Mkrn1 antibody and tERK was used as a loading control. (C–F) Bright-field images of whole ovaries from control (C), *Mkrn1*^*exS*^ (D), *Df(3L) BSC418/Mkrn1*^*exS*^ (E), and *Df(3L) BSC419/Mkrn1*^*exS*^ flies. (G, H) Confocal images of ovariole immunostaining from control (G) and *Mkrn1*^*exS*^ (H) flies. Dissected ovaries were stained for Vasa (green), Actin (red, phalloidin), and the DNA (blue, hoechst). Please note that *Mkrn1*^*exS*^ egg chambers did not progress to vitellogenic stages and eventually degenerated from stage 7 resulting in egg chambers with only germline cells (arrow). (I, J) Ovaries were immunostained for DNA (blue, top panel) and Mkrn1 (red, bottom panel) from control (I) and *Mkrn1*^*exS*^ (J) flies. Mkrn1 was ubiquitously observed in cytoplasm of follicle cells, nurse cells, and oocytes throughout oogenesis, and Mkrn1 was enriched in the pole plasm (arrow head) in the control. Absence of Mkrn1 detection in *Mkrn1*^*exS*^ ovaries validates the gene knockout and specificity of the Mkrn1 antibody. Numbers above each egg chamber indicate the developing stage of ovarioles. Scale bars = 20μm.

*Mkrn1*-null flies (*Mkrn1*^*exS*^,[37]) are completely viable with no obvious phenotype. However, we found that the female flies were sterile whereas the male flies were fertile. This was surprising because our previous study did not reveal sexual dimorphism of *Mkrn1*-mutant flies during pupariation and *Mkrn1* is expressed in prothoracic endocrine glands [37]. To determine the role of *Mkrn1* in female reproduction, we first examined *Mkrn1* mRNA levels in ovaries. We found that *Mkrn1* mRNA expression was highly enriched in ovaries compared to the head, thorax, and abdomen (Fig 1A), which is consistent with our observed phenotype of *Mkrn1*-mutant female sterility. Next, we analyzed Mkrn1 protein levels using anti-Mkrn1 antibody and further confirmed that it is highly enriched in ovaries compared to other tissues, suggesting an important role of *Mkrn1* in *Drosophila* female fertility (Fig 1B).

### Vitellogenesis did not occur in *Mkrn1*-null ovaries

The ovaries of *Mkrn1*^*exS*^ flies were much smaller than control ovaries and did not contain mature eggs (Fig 1C and 1D). To confirm that the female sterility phenotype was truly due to loss of function of the *Mkrn1* gene, we crossed *Mkrn1*^*exS*^ with two different deficiency lines uncovering this locus for the complementation testing. Female heterozygotes of both strains, *Df(3L)BSC418*/*Mkrn1*^*exS*^ and *Df(3L)BSC419*/*Mkrn1*^*exS*^, were sterile. Moreover, ovarian phenotypes and size were indistinguishable among the *Df(3L)BSC418*/*Mkrn1*^*exS*^, *Df(3L)BSC419*/*Mkrn1*^*exS*^, and *Mkrn1*^*exS*^ homozygotes, confirming that this allele is the true null allele *of Mkrn1* (Fig 1E and 1F).

To examine the defects of oogenesis more closely, ovaries were stained with phalloidin and antibody against VASA protein. All egg chambers were previtellogenic and lacked the dramatic morphological defects apparent in egg chambers prior to the previtellogenic stage (Fig 1G and 1H). Oogenesis did not proceed after stage 7. After several strings of stage 7 egg chambers, the follicle cells degenerated, resulting in egg chambers with only germline cells. Because *Mkrn1* deficiency displayed stage-dependent defect, we examined if *Mkrn1* had a stage-specific expression pattern using the Mkrn1 antibody. We first confirmed that our antibody recognized the Mkrn1 protein by immunofluorescence staining of ovaries since Mkrn1 staining disappears in *Mkrn1*^*exS*^ (Fig 1J). *Mkrn1* was ubiquitously expressed in follicle cells, nurse cells, and oocytes throughout oogenesis (Fig 1I).

### Cell-autonomous role of *Mkrn1* was displayed in germline cells

Hormone signaling dysfunction, such as impaired insulin or ecdysone signaling pathways, can cause female sterility with vitellogenesis defects in flies [12, 24]. Although the previous study suggests a role of *Mkrn1* in neuroendocrine cells in controlling the timing of pupariation [37], ovary-enriched expression of *Mkrn1* and female specific sterility suggest that *Mkrn1* function is autonomously required for normal ovarian function. To test this, we performed a mosaic clonal analysis using FLP-FRT mitotic recombination and examined mutant clones in the ovaries. This experiment also made it possible to further dissect the specific cell types that required *Mkrn1* function in the ovaries.

**Fig 2:**
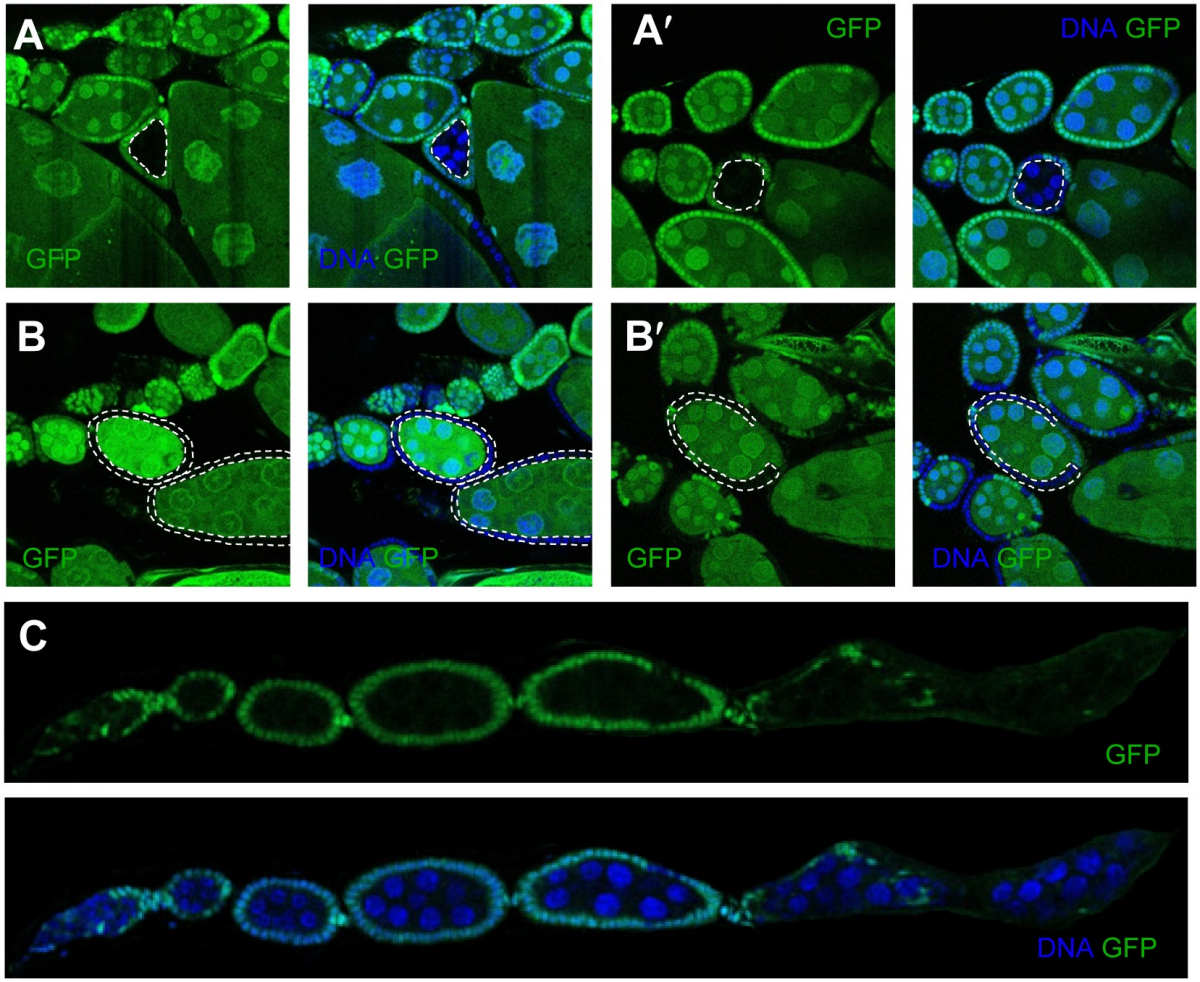
*Mkrn1* function was required in germline cells, but not follicle cells, for egg chamber growth. (A, B) *Mkrn1*^*exS*^ cells are marked by the absence of GFP (green). Posterior egg chambers consisting of *Mkrn1*^*exS*^ germline cysts and wild-type follicle cells were abnormally smaller than the anterior egg chambers (A and A′). Egg chambers with wild-type germline cysts and mutant follicle cells grew normally and proceeded to vitellogenic stages (B and B′). (C) Ovarioles with all egg chambers had *Mkrn1*^*exS*^ mutant germline cysts and wild-type follicle cells phenocopied the *Mkrn1*^*exS*^ mutant. The dashed line indicates the *Mkrn1*-null clones.

Interestingly, the egg chambers with *Mkrn1*-mutant germline cysts enclosed by wild-type follicle cells were smaller than its anterior cysts, which was abnormal because oogenesis proceeds serially, resulting in larger posterior than anterior egg chambers (Fig 2A and 2A′). The smaller size of *Mkrn1*^*exS*^ germline cysts suggests that *Mkrn1* autonomously regulates germline cyst growth. In contrast, the egg chambers with wild-type germline cells enclosed by *Mkrn1*^*exS*^ follicle cells did not exhibit phenotypic differences from neighboring wild-type egg chambers. Even when all of the follicle cells of the egg chamber were *Mkrn1* mutants, if germline cells were wild-type, the egg chambers developed normally and reached vitellogenic stages suggesting that the loss of *Mkrn1* in follicle cells is not responsible for the oogenesis defect (Fig 2B and 2B′). We also found that ovarioles consisting of egg chambers with *Mkrn1* mutant germline cysts enclosed by wild-type follicle cells exhibited exactly the same phenotype as *Mkrn1*^*exS*^ ovarioles (Fig 2C). *Mkrn1*—null germline cyst development did not proceed past stage7 before the egg chamber degenerated. From these results, we concluded that the function of *Mkrn1* is autonomous in ovaries, more specifically in germline cells.

### *Mkrn1*-mutant ovaries did not display aberrant Notch signaling during mid-oogenesis

The ME switch occurs during mid-oogenesis. This transition is a prerequisite for egg chambers to enter vitellogenesis, and Notch signaling regulates this transition around stage 6 [5–7].

**Fig 3:**
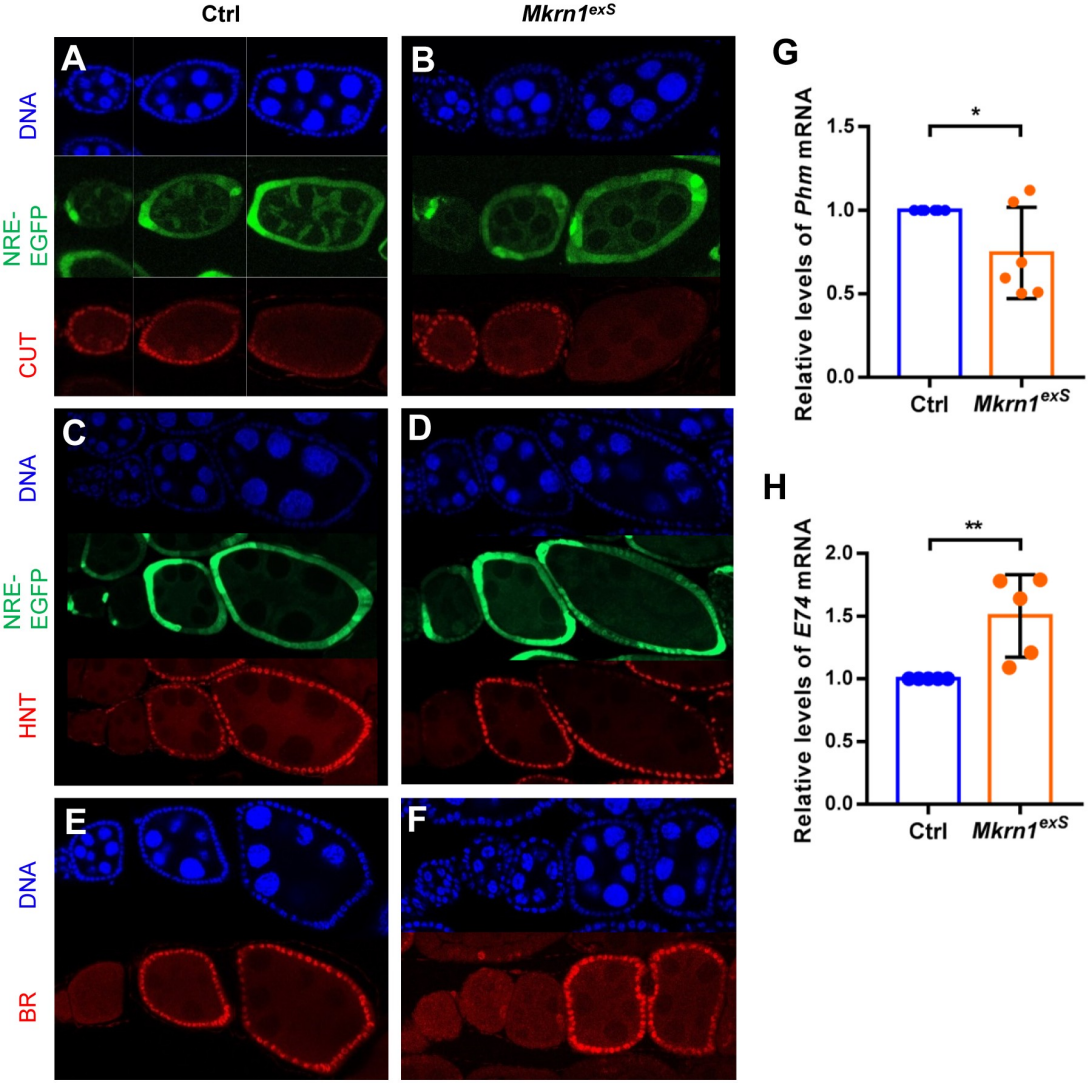
Expression of Notch targets and ecdysone-responsive genes were not affected in *Mkrn1*-null *Drosophila* ovaries. (A–F) Egg chambers from control and *Mkrn1*^*exS*^ flies were stained for Cut (A and B), Hnt (C and D), and Br (E and F) (shown in red). (A–D) NRE-EGFP (GFP) was used as a Notch signaling reporter and was activated around stage 6 in both the control and *Mkrn1*^*exS*^ ovaries. Cut was expressed up to stage 6 and Hnt was activated around stage 6 in both control and *Mkrn1*^*exS*^ ovaries. Br was also activated after stage 6 in both the control and *Mkrn1*^*exS*^ ovaries. (G and H) Control and *Mkrn1*^*exS*^ ovaries were dissected from one-day-old females. Quantitative real-time PCR was performed to measure mRNA levels of *Phm* (G) and *E74* (H). Relative mRNA levels are shown and error bars represent SEM from five independent experiments. Asterisks indicate statistically significant differences (Student’s t-test: *P < 0.05; **P < 0.01).

Because *Mkrn1* mutants exhibit terminated development at the stages immediately after the ME switch, we examined if Notch signaling was affected in *Mkrn1*^*exS*^ flies. Notch regulates the expression of transcription factors such as *cut*, *hindsight (hnt)*, *and broad (br)* during the ME switch. We introduced Notch reporter NRE-EGFP, in which Notch response element drives expression of EGFP, into control and *Mkrn1*^*exS*^ flies. NRE-EGFP expression was stronger around stage 6. The onset pattern of NRE-EGFP expression was indistinguishable in control and *Mkrn1*^*exS*^ flies (Fig 3A–3D). We also examined three well-known Notch targets: *cut*, *hnt*, and *br* to determine Notch activity. When Notch signaling is activated around stage 6, *cut* expression is repressed and *hnt* and *br* expression is induced. In *Mkrn1* mutants, all three Notch targets showed similar expression patterns compared to the control (Fig 3A–3F). Our results showed that *Mkrn1* did not affect the onset of Notch signaling in follicle cells during the ME switch.

### Ecdysone signaling was not affected in *Mkrn1*-null *Drosophila* ovaries

In *Drosophila* ovaries, ecdysone signaling is cell-autonomously required in germline cells for proper development. Ovaries display previtellogenic egg chambers without appropriate ecdysone signaling, as shown in ecdysone receptor (*EcR*) and ecdysoneless cell cycle regulator (*ecd*) mutants, which are similar to the *Mkrn1*^*exS*^ phenotype [24, 25]. We previously showed that ecdysone synthesis was down-regulated in prothoracic glands during larval development in *Mkrn1*^*exS*^ larvae [37]. Thus, we tested if reduced ecdysone signaling was responsible for the oogenesis defects in *Mkrn1*^*exS*^ ovaries. In adults, ovaries can also produce ecdysone [39], We measured mRNA levels of *phantom* (*phm*), an enzyme required for ecdysone synthesis, and found its transcript levels to be slightly reduced in *Mkrn1*^*exS*^ ovaries compared to controls (Fig 3G). These data are consistent with what was observed in *Mkrn1*^*exS*^ larvae, suggesting the mechanism by which Mkrn1 regulates ecdysone synthesis could be preserved in ovaries. Next, as a surrogate indicator for ecdysone signaling, the expression of the early ecdysone response gene, *E74*, was examined by quantitative real-time PCR. *E74* transcript levels were reduced in *Mkrn1*^*exS*^ larvae [37]; however, its expression levels were 1.5-fold higher in *Mkrn1*^*exS*^ ovaries. Thus, the reduction of ecdysone synthesis did not appear to significantly affect ecdysone signaling transduction (Fig 3H), presumably due to the ecdysone synthesized by other tissues [39–41]. Moreover, *br*, another target of ecdysone signaling [5, 42], was not affected in *Mkrn1*^*exS*^ ovaries, further supporting this postulation (Fig 3E and 3F). On the other hand, excessive ecdysone signaling can induce apoptosis of nurse cells at stage 8 and 9 [43]. Although *Mkrn1*^*exS*^ ovaries showed a slight increase in ecdysone signaling, egg chambers remained intact following stages 8–9 and nurse cells did not undergo apoptosis, as shown by the absence of condensed nuclei; therefore, the increased ecdysone signaling was not the cause of the ovarian defects observed in *Mkrn1*^*exS*^ flies.

### Insulin signaling was significantly reduced in *Mkrn1*-mutant cysts

**Fig 4:**
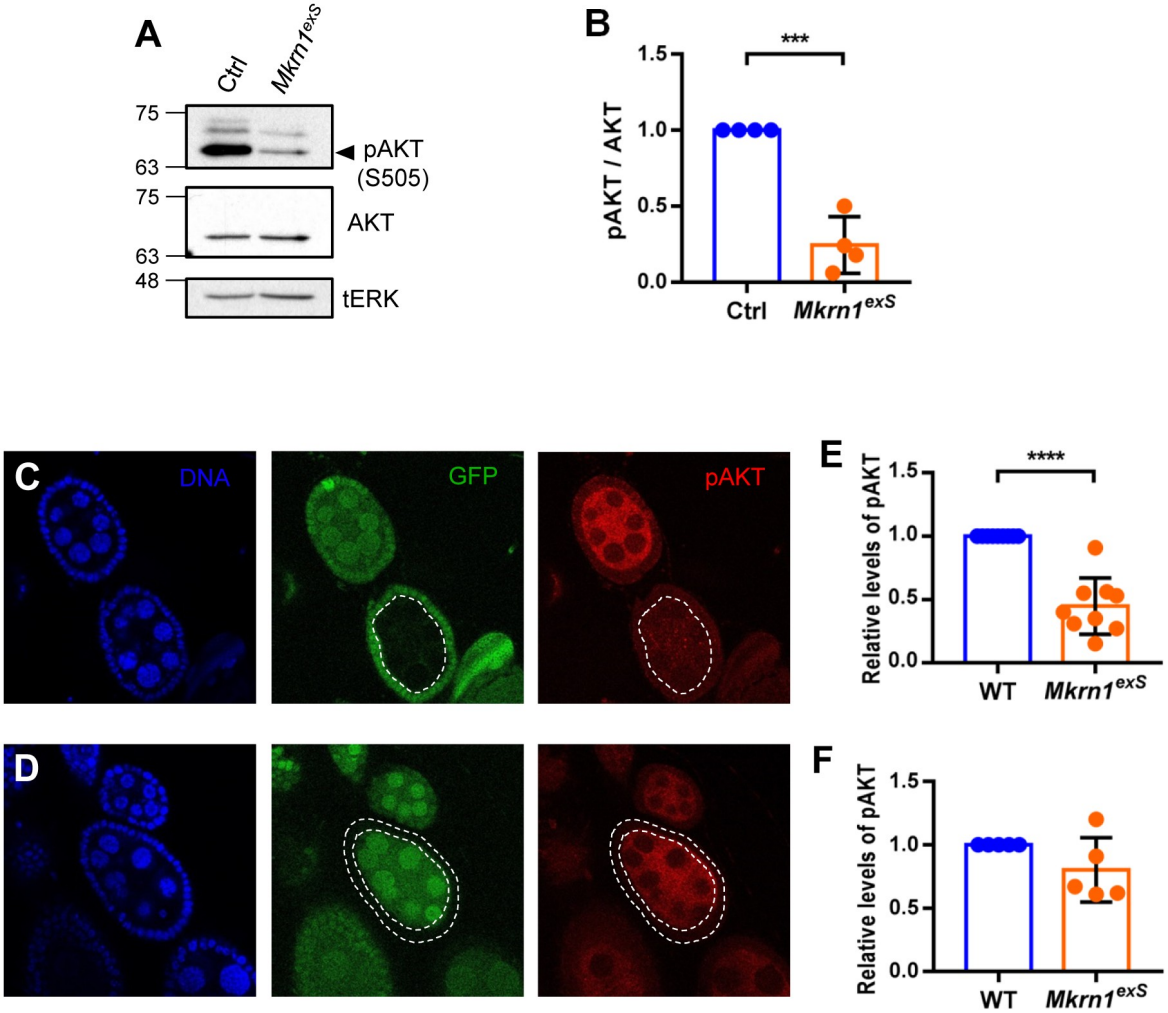
p-AKT levels were reduced in *Mkrn1*^*exS*^ ovaries. (A) Protein extracts were prepared from ovaries of one-day-old control and *Mkrn1*^*exS*^ female flies and analyzed by western blot using anti-p-AKT and anti-AKT antibodies, and tERK as the loading control. (B) Relative p-AKT levels were quantified by measuring band intensities and normalized by total AKT protein levels. Error bars represent SEM from four independent experiments. Asterisks indicate statistically significant differences (Student’s t-test: ***P<0.001). (C–F) Confocal images of immunostaining for p-AKT (red) and DNA (blue) of ovaries from *Mkrn1*^*exS*^flies containing mutant germline and follicle cells. *Mkrn1*^*exS*^ cells are distinguished by the absence of GFP (green). p-AKT level were significantly reduced in *Mkrn1*^*exS*^ germline cells, but not in *Mkrn1*^*exS*^ follicle cells. Fluorescence intensities of p-AKT were quantified and *Mkrn1*^*exS*^ germline cysts were compared with similarly sized neighboring wild-type germline cysts. *Mkrn1*^*exS*^ follicle cells were quantitated and compared to nearby wild-type follicle cells. Error bars represent SEM for 10 germline clones and five follicle clones. Asterisks indicate statistically significant differences (Student’s t-test: *P < 0.05; ****P < 0.0001).

The mid-oogenesis check point is the stage right before the onset of vitellogenesis. In addition to Notch and ecdysone signaling pathways, insulin and TOR signaling pathways are also essential to ensure that there are enough nutrients for the egg production progression. InR hypomorphs show very similar phenotypes to *Mkrn1*^*exS*^ ovaries in that they are both previtellogenic [12]. The similarity of ovarian phenotypes of *Mkrn1*^*exS*^ and insulin signaling pathway mutants led us to examine the role of *Mkrn1* in the insulin signaling pathway. We used phosphorylated AKT (p-AKT) as a readout of insulin signaling pathway activation. p-AKT levels were measured by western blot analysis of ovarian protein extracts from one-day-old female flies to ensure that both control and mutant flies had similar sized ovaries at the same stages of oogenesis for proper molecular comparison. We found that AKT phosphorylation was greatly reduced in *Mkrn1*^*exS*^ ovaries compared to control, indicating that insulin signaling is reduced in *Mkrn1*^*exS*^ ovaries (Fig 4A and 4B).

As described earlier, *Mkrn1* function is essential for germline cyst, but not follicle cell, growth. If this growth defect is due to decreased insulin signaling, we could expect different influences of *Mkrn1* on various cell types. To determine if there are cell-type specific differences in insulin signaling, ovaries were immunostained for p-AKT. Levels of phosphorylated AKT were significantly reduced in *Mkrn1*^*exS*^ germline cells and there was no difference in *Mkrn1*^*exS*^ follicle cells (Fig 4C–4F). This result is consistent with the growth-limiting phenotypes of *Mkrn1*^*exS*^ only manifested in germline cells but not in follicle cells. Reduction of insulin signaling in germline clones of *Mkrn1*^*exS*^ implies that Mkrn1 function is required for insulin signaling and normal development of ovaries.

### *Mkrn1* levels depend on nutrient availability and TOR signaling

**Fig 5:**
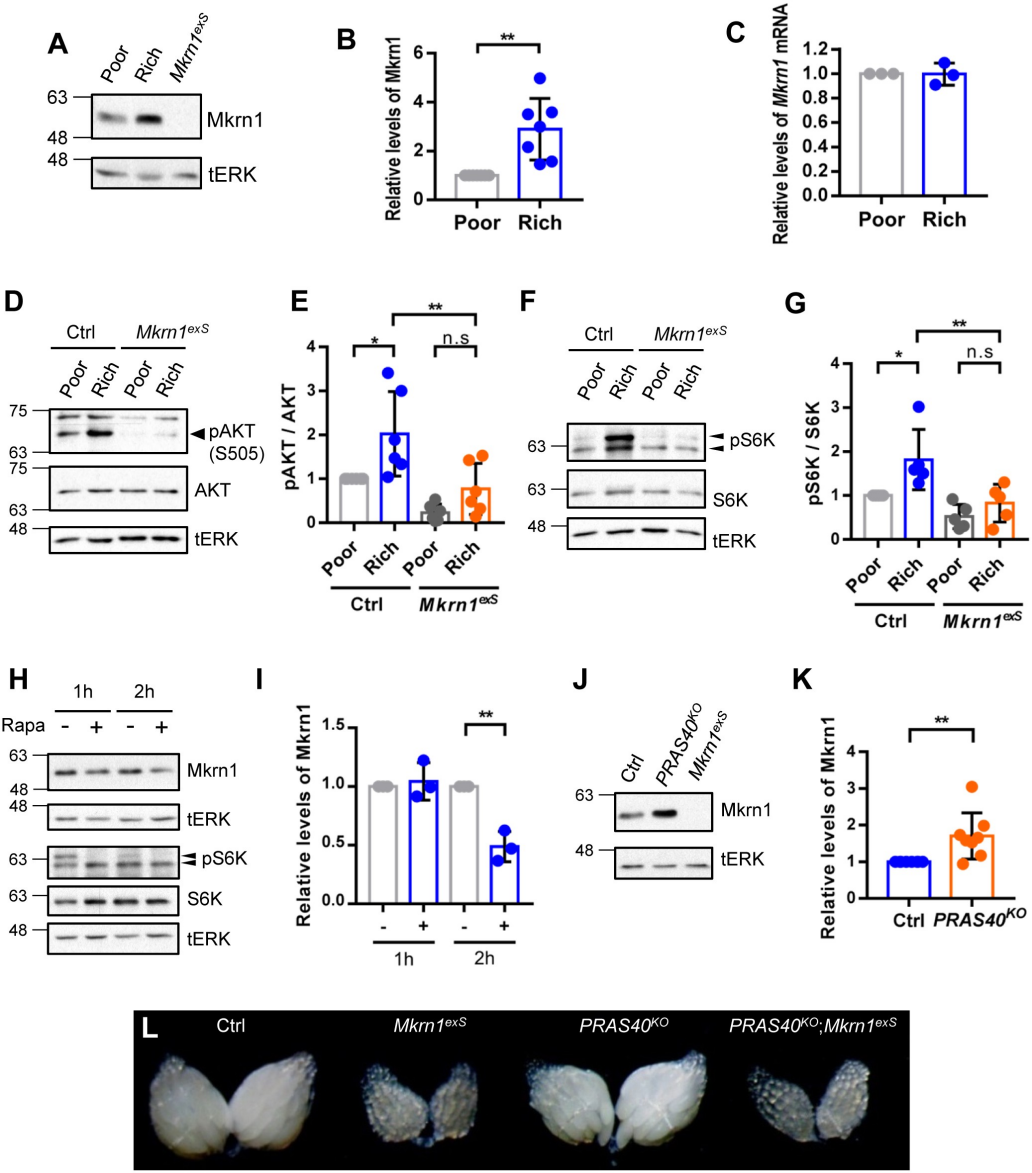
Mkrn1 ovarian protein levels are regulated by nutritional status and TOR signaling in Drosophila. (A) Newborn female flies were reared for 24 hours under the indicated nutritional conditions. Protein extracts from flies under nutrient-poor or -rich conditions were subjected to immunoblot analysis using anti-Mkrn1 antibody, and tERK as the loading control. (B) Mkrn1 protein levels were quantified by measuring band intensities and the relative levels are shown. Error bars represent SEM from seven independent experiments. Asterisks indicate statistically significant differences (Student’s t-test: **P<0.0.01). (C) Ovaries from flies under the same nutrient conditions were used for mRNA extraction. Quantitative real-time PCR was performed to measure *Mkrn1* mRNA levels and the relative levels are shown. Error bars represent SEM from three independent experiments. (D-G) Control and *Mkrn1*^*exS*^ females were reared under nutrient-poor or -rich conditions for 24 hours. Protein extracts were then obtained from the ovaries and subjected to immunoblot analysis using anti-p-AKT and anti-AKT antibodies (D), anti-pS6K and anti-S6K (F) and tERK as the loading control. Relative levels of p-AKT (E) and pS6K (G) were quantified by measuring band intensities and normalized to the total AKT protein levels (E) and S6K protein levels (G). (H and I) The ovaries were dissected and incubated in PBS with or without rapamycin (1μM) for the indicated time. Protein extracts from the ovaries were subjected to immunoblot analysis using anti-Mkrn1 antibody and tERK as the loading control. Relative levels of Mkrn1 were determined as described above. The error bars represent SEM from three independent experiments and the asterisks indicate statistically significant differences (Student’s t-test: **P<0.01). (J and K) Ovaries from control and *PRAS40*^*KO*^ female flies were dissected and subjected to western blot analysis of the Mkrn1 levels using anti-Mkrn1 antibody and tERK as the loading control. Relative levels of Mkrn1 were determined as described above. The error bars represent SEM from eight independent experiments and the asterisks indicate statistically significant differences (Student’s t-test: *P<0.05, **P<0.01). (L) Bright filed images of ovaries from control, *Mkrn1*^*exS*^, *PRAS40*^*KO*^, and *Mkrn1*^*exS*^ *PRAS40*^*KO*^ flies are shown.

The ovarian phenotype of *Mkrn1*^*exS*^ is very similar to those of nutrient signaling pathway hypomorphs as well as observations under starvation conditions. To test if Mkrn1 protein levels are regulated by nutrient availability, flies were subjected to nutrient-poor or -rich diet conditions for 1 day. Because we wanted to focus on signaling events in early stages of egg chambers, we used one-day-old females for the analysis. Protein extracts from ovaries of female flies, maintained in either nutrient-poor or -rich diet conditions, were examined by western blot analysis. We found that Mkrn1 protein levels were significantly reduced in ovaries of starved flies (Fig 5A and 5B). To determine if this reduction in Mkrn1 protein levels was due to reduced transcription, we performed quantitative real-time PCR for *Mkrn1* mRNA transcript levels; *Mkrn1* mRNA levels were not affected by nutritional status (Fig 5C). Thus, Mkrn1 is regulated by nutritional status at the post-translational level. To examine how Mkrn1 affects insulin and TOR signaling in ovaries depending on nutritional status, we examined the levels of p-AKT (Fig 5D and 5E) and p-S6K (Fig 5F and 5G), downstream effector of insulin and TOR signaling, in control and *Mkrn1*^*exS*^ ovaries under nutrient-poor and -rich conditions. We found that both p-AKT and p-S6K signals were significantly increased by a nutrient-rich diet in control flies but not in *Mkrn1*^*exS*^. Moreover, the levels were very low compared to control flies under both nutrient conditions. This indicates that *Mkrn1* is an important modulator of the insulin and TOR signaling pathway that holds the ability to respond to nutritional status signals.

Because Mkrn1 level were increased by a nutrient- rich diet, we sought to determine if TOR signaling regulates Mkrn1 protein levels. First, we treated ovaries from one-day-old females with rapamycin, a TOR inhibitor. Compared to vehicle treated ovaries, Mkrn1 levels were reduced in rapamycin treated ovary (Fig 5H and 5I). Because PRAS40 functions as an ovary-specific negative regulator of TORC1 in *Drosophila* [23], we also examined MKRN1 levels in *PRAS40* mutant ovaries, and found that Mkrn1 levels were increased in the ovaries from *PRAS40*^*KO*^, *PRAS40* null mutants (Fig 5J and 5K). These data confirmed that Mkrn1 levels are regulated by TOR signaling. A prior report indicated that PRAS40 acts downstream of insulin signaling in the ovaries and rescued the sterile phenotype observed in *chico*^*1*^ mutants [23]. To test whether PRAS40 could also rescue the phenotype observed in *Mkrn1*^*exS*^, we generated double mutants for *PRAS40* and *Mkrn1*. In contrast to the *chico*^*1*^ mutant, the introduction of the *PRAS40* mutant to *Mkrn1*^*exS*^ did not rescue the sterility observed in *Mkrn1*^*exS*^ females. In addition, the ovaries from the double mutants were indistinguishable from the ovaries obtained from *Mkrn1*^*exS*^ females, suggesting that *Mkrn1* is epistatically downstream of *PRAS40* (Fig 5L), which was consistent with the finding that MKRN1 levels were elevated in the absence of PRAS40. Taken together, the enrichment of Mkrn1 in the ovaries suggests that Mkrn1 acts as a tissue-specific factor that is regulated by a protein-rich diet through TOR signaling and that Mkrn1 regulates the insulin/Tor signaling pathway to drive oogenesis only in the presence of sufficient nutrients.

## Discussion

In this study, we revealed a previously unknown role of *Mkrn1* in oogenesis. *Mkrn1* loss leads to previtellogenic ovaries, which are also observed in hypomorphic models of nutrient-dependent signaling pathway components. Nutrient-dependent control of signaling pathways is especially important for the onset of vitellogenesis because the following states require high amounts of energy for protein synthesis. Our data showing *Mkrn1* as a positive regulator of the insulin signaling pathway is consistent with its mutant phenotype.

Clonal analysis of the *Mkrn1* mutation revealed that Mkrn1 is required in germline cells, but not in the follicle cells in egg chambers. Reduction of p-Akt was only observed in germline cysts, but not in follicle cells. These results contrast with the ubiquitous expression of Mkrn1 in ovaries and the preferential degeneration of follicle cells in *Mkrn1* null mutants. The degeneration of follicle cells also occurred in ovarioles consisting of wild-type follicle cells and *Mkrn1* mutant germline cells (Fig 2C), indicating a non-autonomous role of *Mkrn1* in the degeneration of neighboring follicle cells. A Similar phenotype was described as ‘peas without pods’ (Pwop) in egg chambers harboring insulin and TOR signaling pathway mutant germline clones, such as InR hypomorph, chico^1^, and S6K mutants [44]. The similarity between *InR*, *chico*, *S6K*, and *Mkrn1* mutant germline clones suggests that Mkrn1 has a regulatory role in insulin signaling in germline cells. Collectively, we think that germline cells might be protected from degeneration induced by a reduction in insulin signaling caused either by the *Mkrn1* mutation or by other components of the insulin signaling pathway, but not the follicle cells.

Unlike key components of the insulin signaling pathway, *Mkrn1* expression is highly enriched in ovaries. This explains why *Mkrn1*^*exS*^ flies do not exhibit alterations in body size while *chico*, *lnk*, and *InR* hypormorphs exhibit a reduction in body size in addition to the female-sterile phenotype. This implies that *Mkrn1* is a tissue-specific modulator of the insulin signaling pathway. However, the significance of *Mkrn1* enrichment in ovaries remains unclear. According to our current study, as well as others, we can infer that tissue-specific differences in the dynamics and strength of insulin signaling is required based on the organ for proper homeostasis. For example, in developing flies, brain growth is spared during nutrient restriction. This tissue-specific effect is achieved by strong *Alk* expression in the brain, which regulates the TOR and insulin signaling pathways [45]. In adult male flies, the genitals are also resistant to starvation. This is made possible by decreasing the expression FOXO in this tissue to ensure the insulin signal is not inhibited by nutrient restriction [46]. Conversely, ovaries are immediately wasted during starvation to preserve energy for survival and are particularly sensitive to the availability of amino acids [8].

Ovary-specific factors or mechanisms are required to trigger events that are restricted to the ovaries by ubiquitous nutritional changes or by the genetic manipulation of insulin signaling. A prior study of *Drosophila* PRAS40 supports the tissue specific regulation of the nutrient signaling pathway [23]. The authors showed that an ovary specific connection existed between the insulin and TOR signaling pathways. Although PRAS40 is ubiquitously expressed, the loss of PRAS40 function only resulted in enlarged ovaries with increased p-S6K levels, but a normal body size. PRAS40 acts downstream of insulin signaling and specifically regulates TORC1 activities in the ovaries. Our data showed that PRAS40 negatively regulates Mkrn1 levels and that Mkrn1, in turn, positively regulates insulin/Tor signaling. Hence, because it is highly expressed in the ovaries, regulated by nutritional status and TOR signaling, and positively regulates the insulin/Tor signaling pathway, we propose that *Mkrn1* is a nutrient-dependent, tissue-specific regulator of insulin/Tor signaling in the ovaries. Tissue-specific modulators of insulin signaling account for the different responses of organs in mammals as well. Prior studies have shown reduced insulin sensitivity in the ovaries of lean mice, and increased insulin sensitivity in the ovaries of obese mice compared to peripheral metabolic tissues. This could be due to enhanced regulation of insulin signaling by *Irs-1* and *Irs-2* in the ovaries compared to only *Irs-1* regulation in the periphery [21]. To this end, a prior study demonstrated that the deletion of *Irs-2* caused female infertility in mice [20].

The question of how Mkrn1 regulates insulin signaling in response to nutritional status thus remains. The potential molecular function of Mkrn1 as a ubiquitin E3 ligase has been studied. The Mkrn1 substrates identified include p53, p21, PPARγ, hTERT, Pten, APC, and AMPK [31–36]. Among these substrates, AMPK and Pten are regulators of energy-dependent signaling pathways. AMPK was recently identified as a Mkrn1 target [33]. Because AMPK is activated by low cellular-energy levels, it may play a critical role in ovaries by suppressing ovarian growth and development in a dietary-restricted environment. It could be hypothesized that AMPK levels become constitutively high and inhibit anabolic processes and vitellogenesis in *Mkrn1*^*exS*^ flies. However, an energy-dependent role of AMPK would only be critical in follicle cells and dispensable in the germline cells of *Drosophila* ovaries [47]. This would not be consistent with our results that Mkrn1 is essential in germline cells, but not follicle cells, for proper oogenesis. Thus, AMPK does not seem to be responsible for the previtellogenic ovarian phenotype of *Mkrn1*^*exS*^ flies. Pten is a key regulator of the insulin signaling pathway and s destabilized by Mkrn1. Interestingly, *Mkrn1* functions as a positive-feedback regulator of the PI3K/AKT signals in cervical cancer progression because it is stabilized by AKT-mediated phosphorylation and it destabilizes Pten, which leads to further activation of the insulin signaling pathway [34]. Hence, we speculated that Mkrn1 destabilizes Pten in the ovaries and that the increased PTEN levels in *Mkrn1*^*exS*^ flies could account for the decreased level of AKT phosphorylation. However, the absence of a reliable *Drosophila* Pten antibody and the lethal *Pten* mutation phenotype made it difficult for us to test this idea. Thus, whether or not *Mkrn1* regulates the insulin signaling pathway through Pten or other target(s) during oogenesis remains to be determined.

Although our data suggests an important role of Mkrn1 in insulin/TOR signaling during oogenesis, it is still possible that other known targets of Mkrn1 may account for the sterile phenotype observed in *mkrn1* null mutant. For example, APC was recently identified as an Mkrn1 substrate and functions in WNT signaling [36]. In *Drosophila*, WNT signaling is required for stem cell maintenence [48] but its role in mid-oogenesis is unknown. If Mkrn1 destabilizes APC in *Drosophila* ovaries, the increase in APC in *Mkrn1*^*exS*^ would theoretically lead to the degradation of Armadillo, a *Drosophila* homologue of mammalian β-catenin. However, this was not the case in *Mkrn1*^*exS*^. Armadillo levels were increased in *Mkrn1*^*exS*^ compared to controls (S1 Fig) suggesting that APC is not responsible for the *Mkrn1*^*exS*^ female sterility phenotype.

Intriguingly, we observed that Mkrn1 protein is highly enriched in pole plasm in this study (Fig 1I). Pole plasm contains *osk* and *staufen* mRNAs, which are important regulators of germ cell development [49, 50]. It has been previously reported that Mkrn1 is an RNA-binding protein [51–53]. *Mkrn1* was suggested to control protein translation in mammalian neuronal cells [53]. Collectively, our findings along with others raise the possibility that *Mkrn1* may be actively involved in translational control of mRNAs in the pole plasm, thereby regulating the development of germ cell. Although this idea requires further investigation, it does not conflict with the E3 ligase role of Mkrn1 in the insulin signaling pathway. Translational control is ultimately a downstream event of nutrient-dependent signaling, such as the insulin and mTOR pathways, and therefore, should be precisely regulated. *Mkrn1* could perform dual functions as a translational activator and a E3 ligase to better coordinate energy-dependent signaling and subsequent protein translation.

Insulin signaling for the regulation of oocyte development is highly conserved in mammals [16]. Although the hypothalamic-pituitary-gonadal axis of the hormonal system is well established in mammalian reproduction, insulin signaling is also heavily implicated in ovarian development. Challenging situations, such as obesity-induced infertility, can be reversed by modulating insulin signaling [54]. However, precise regulation of insulin signaling is poorly understood. For example, the ovary-specific deletion of *Pten* or *Pdk1* causes premature ovarian failure despite opposite regulatory roles in the insulin signaling pathway, implying the complexity of the mechanism [55, 56]. We suggest that additional positive regulators such as *Mkrn1* and the tissue specific interaction of insulin and TOR signaling may introduce additional complexity and dynamics to the system. Moreover, our study provides new insights into the mechanism underlying the tissue specificity of the insulin signaling pathway and the mechanisms leading to diet-related female fertility.

## Supporting information

S1 Fig Armadillo levels were increased in *Mkrn1*^*exS*^ ovaries. (PDF)
