# Supplementary material for: Makorin 1 is required for Drosophila oogenesis by regulating insulin/Tor signaling
Source: PLoS One. 2019 Apr 22;14(4):e0215688. doi: 10.1371/journal.pone.0215688 (PMC6476528; doi:10.1371/journal.pone.0215688)
Supplement: S1 Fig — (PDF) [file pone.0215688.s001.pdf]

Supplementary figure 1.

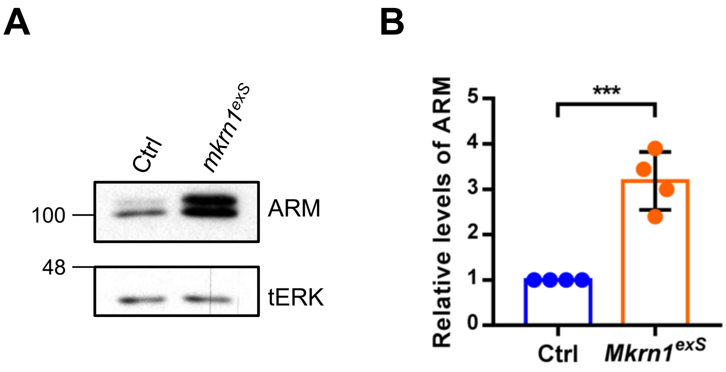

Supplementary Figure 1. Armadillo levels were increased in *Mkrn1^{exS}* ovaries. (A) Protein extracts from ovaries of one-day-old control and *Mkrn1^{exS}* female flies were prepared and analyzed by western blot using anti-Armadillo antibodies, and tERK as the loading control. (B) The Armadillo levels were quantified by measuring the band intensities and relative levels are shown. Error bars represent SEM from four independent experiments. Asterisks indicate statistically significant differences (Student's t-test: ***P<0.001).
